# A Review on the Role of Ethylenediaminetetraacetic Acid (EDTA) in the Treatment and Understanding of Psoriasis

**DOI:** 10.7759/cureus.16424

**Published:** 2021-07-16

**Authors:** Amreen Sunil, Gurneet Shaheed, Akshay J Reddy, Neel Nawathey, Hetal Brahmbhatt

**Affiliations:** 1 Dermatology, California Northstate University College of Medicine, Elk Grove, USA; 2 Integrative Medicine, University of California, Los Angeles (UCLA), Los Angeles, USA; 3 Opthalmology, California Northstate University College of Medicine, Elk Grove, USA; 4 Dermatology, California Northstate University, Rancho Cordova, USA; 5 Psychiatry, Mercy General Hospital, Sacramento, USA

**Keywords:** psoriasis, edta, skin, lipoprotein, immunoanalysis

## Abstract

Psoriasis is a long-term, autoimmune inflammatory condition characterized by red, scaly plaques that can range from a few patches to total skin coverage. Over the past 60 years, and more recently, the metal-chelating agent ethylenediaminetetraacetic acid (EDTA) has proven increasingly useful in the treatment and understanding of psoriasis and related conditions. This review will analyze the current role and effectiveness of EDTA in clinical and non-clinical studies designed to improve the diagnosis and treatment of psoriasis in patients. Currently, EDTA demonstrates great medical benefit in the treatment of psoriasis as an antioxidant and as an inhibitor of beta-lipoprotein production. EDTA additionally functions well in research applications due to its ability to maintain red blood cell structural integrity. The authors find that the perceived impact of EDTA in the understanding and combating of psoriasis to be greatly underestimated and is therefore in need of increased awareness and attention by healthcare professionals, dermatologists, and clinical researchers.

## Introduction and background

Psoriasis is among the most common autoimmune, inflammatory conditions in the world, with adult estimates ranging from as low as 0.91% in the United States to as high as 8.5% in Norway [1]. Currently, psoriasis is believed to be a predominantly genetically related disease but certain environmental factors including stress, obesity, and smoking have been shown to trigger and exacerbate the condition [2]. Along with the immediate effects of psoriasis such as dryness, itchiness, and inflammation, patients with psoriasis have a higher risk of developing cancer and cardiovascular diseases. 

Psoriasis can present itself in various forms, most commonly as plaque psoriasis but also as pustular, inverse, napkin, guttate, oral, and erythrodermic forms. Plaque psoriasis, comprising about 90% of psoriasis patients, typically appears as inflamed red skin lesions covered with silvery-white, scaly skin [3]. Other forms of psoriasis, though much rarer, vary in severity and tend to be characterized by location. Inverse psoriasis, for example, is traditionally diagnosed by the appearance of smooth, inflamed patches on skin folds such as the armpit or groin [3]. Erythrodermic psoriasis, alternatively, is the most severe presenting as widespread inflammation across nearly 90% of the body and is often accompanied by severe dryness, itching, swelling, and pain [3]. 

Although there is no current cure for this condition, topical corticosteroids have proven to be the most effective treatment of psoriasis when used for eight weeks continuously [2]. Corticosteroids are generally used to treat psoriasis in combination with vitamin D analogues and phototherapy, they are rarely used as the sole agent to treat psoriasis [2]. This treatment option is not ideal as there are many complications that could arise from the excessive use of corticosteroids. In order to help psoriasis patients handle their symptoms without dealing with several unwanted side effects, different drugs were developed in order to help combat the disease. In fact, several biologics were created in order to remove psoriatic plaques and target inflammatory pathways that were being affected by the disease, but the effects of these drugs were short lived and never proved to serve as a mechanism to help patients overcome the disease [2]. Fortunately, methods to better understand psoriasis have improved over time and will be discussed further in this review.

## Review

Psoriasis is an autoimmune skin condition that results in red and scaly patches on the skin. Psoriasis is thought to be caused by genetic factors with some environmental influence. Although there are no cures to this long-term condition, various methods exist to manage the symptoms better. Over the past 60 years, scientists have been trying to better understand these treatments for psoriasis by using the chemical agent ethylenediaminetetraacetic acid (EDTA). EDTA is a metal-chelating agent and has been helpful in patients with heavy-metal poisoning such as lead or mercury [4]. EDTA as a chelating agent is often used in reducing non-specific serum protein interactions in enzyme immunoassays [5]. EDTA is also helpful in creating blood/plasma samples due to its ability to prevent agglutination, however, it's primarily used in immunoanalysis to detect CD3 cells [6]. Psoriasis is an autoimmune disorder which means that certain components of a patient’s immune system are either severely lacking or compromised [3]. In order to determine the severity of the immune complications caused by psoriasis, it is necessary to analyze the concentration of various immune cells, including CD3 cells, within a patient’s body. CD3 cells are particularly important to keep track of as there is a significant positive correlation found between levels of IL-17A, TNF-α, and IL-21 production and CD3+ T cells with Psoriasis Area and Severity Index scores [7]. This data suggests that CD3+T cells may play a role in the pathogenesis of psoriasis. EDTA is also used with combination agents in order to activate the immune cells in the blood samples [8-12]. Generally, these combination agents are either buffers or ionic salts. Combination agents were a necessity in order to activate immune cells. In order to gain a thorough understanding of how the immune cells function within patients who have this disease, it is necessary to conduct immunoanalysis on these cells when they are in the active form. Therefore, a majority of the studies in this review did employ the use of combination agents [8-11,13-20]. 

Additionally, EDTA can also be used to analyze specific enzymes from the biochemical pathways that hinder or progress psoriasis. The epidermis of psoriatic lesions glycogen synthase’s activity is increased four-fold and total phosphorylase activity is only slightly increased. EDTA is used to stabilize glycogen synthase I and D extracted from human epidermis; without EDTA glycogen synthase form I increases due to endogenous phosphatases and dephosphorylating glycogen synthase D [15]. EDTA is also often used to stabilize blood samples in order to allow for genotyping and enzyme analysis. For example, the eNOS gene was genotyped using EDTA blood samples in order to determine the correlation between the eNOS Glu298Asp polymorphism and the risk of hypertension among psoriatic patients [21]. A total of six articles within the review used EDTA in a similar manner to stabilize blood samples in order to conduct immunoanalysis [8-12,16]. This demonstrates how EDTA is necessary in order to maintain the structure of blood cells so that scientists can accurately determine exactly how psoriasis affects the immune system. Without using EDTA to preserve the integrity of the blood cells, obtaining immunologic data on psoriasis might have proven to be much more difficult. 

EDTA has been used for a variety of aspects that have given medical practitioners more information on how to treat and analyze psoriasis. Some scientists have utilized EDTA as an antioxidant to help combat psoriasis [19-20,22]. Other scientists have used EDTA as a means of attempting to understand psoriasis on an enzymatic, genetic, and immunological level [8-12,16,23-24]. Within the review, in most of the older studies, EDTA was used as an agent to analyze psoriasis on a molecular level. In fact, among the articles within the review that were written prior to 2011, there was only one article that discussed the potential of using EDTA as a means of treating psoriasis [25]. The majority of the literature that discussed using EDTA as an antioxidant to treat psoriasis was produced more recently. This is most likely due to the fact that antioxidants only came to the public’s attention in the 1990s when the effects of free radicals were found to be involved in several chronic conditions such as cancer [26]. When there is an imbalance between oxidants and antioxidants, with more oxidants, oxidative stress occurs. This imbalance can be dangerous and can lead to disruptions in redox signaling and molecular damage. Oxidative stress is involved in psoriasis pathogenesis and affects dendritic cells, T lymphocytes, keratinocytes, and inflammatory signaling [26]. Skin is especially susceptible to reactive oxygen species (ROS), due to the environment and skin metabolism. An increased number or duration of free radicals can override the defense mechanisms present and lead to conditions such as psoriasis. This increased attention to EDTA resulted in studies of EDTA used as an antioxidant occurring in more recent years. The review contained three studies where EDTA was used as an antioxidant in order to treat psoriasis [19-20,22]. Another study in the review utilized EDTA as a chemical treatment in order to cause a decrease of beta lipoproteins [25]. Due to the fact that psoriasis is an autoimmune disease, patients with the condition often have increased levels of beta lipoproteins, and the use of EDTA as a treatment could help reduce these levels [27]. Beta-lipoproteins are lipoproteins that are used to transport cholesterol in the blood (such as low-density lipoproteins or LDL). LDL has a large amount of cholesterol and lower amounts of protein and high levels are associated with heart disease due to the increased cholesterol levels [27]. Therefore finding a cure for psoriasis could potentially lead to a decrease in the high number of cardiovascular deaths that occur each year. Based on the chemical therapy articles in the review, the data could suggest that although EDTA is a great antioxidant, its role in decreasing the concentration of beta-lipoproteins is what is mainly causing the cessation of psoriasis symptoms in patients. 

## Conclusions

Based on the reviewed studies previously mentioned, it is clear that EDTA shows great promise in advancing scientific understanding of psoriasis and related conditions. In addition, EDTA’s ability to reduce beta-lipoprotein concentrations in patients has demonstrated great value and effectiveness in the treatment of psoriasis. It is extremely crucial to find a cure for psoriasis as it could help prevent the deaths of several patients with cardiovascular issues. Further studies should be conducted analyzing the long-term effects of EDTA as a treatment for psoriasis. Future research utilizing EDTA for immunoanalysis of psoriasis should be conducted in order to further scientific understanding of the disease. Increased attention, however, must be given to EDTA by dermatologists and other practitioners as a potential and viable treatment for psoriasis and related disorders.
